# Equipping pharmacists to improve osteoporosis management for aged care residents: a pilot study

**DOI:** 10.1080/20523211.2026.2669623

**Published:** 2026-05-21

**Authors:** Catherine Laird, Kylie A. Williams, Helen Benson

**Affiliations:** Graduate School of Health, University of Technology Sydney, Sydney, Australia

**Keywords:** Osteoporosis, fracture, pharmacists, aged care

## Abstract

**Background:**

Osteoporosis is a common yet poorly managed condition among aged care residents. This suboptimal management results from medication-related issues, including undertreatment, inappropriate medication choice, and inadequate monitoring. Pharmacists working in aged-care settings have expressed a desire to improve osteoporosis management. However, gaps in their knowledge about osteoporosis management for aged care residents hinder their ability to do so. Providing pharmacists with education on best practices for osteoporosis management could help address this gap. This study pilots an osteoporosis management education initiative for aged care pharmacists and assesses its acceptance and impact on clinical practice.

**Methods:**

An education initiative, based on Australian clinical guidelines and consensus recommendations for osteoporosis management of aged care residents, was developed and delivered to pharmacists. The pharmacist's acceptance of the initiative and its impact on clinical practice were evaluated through a questionnaire, focus groups, and content analysis of medication review reports completed pre- and post-attending the education initiative.

**Results:**

The education initiative was delivered to 33 pharmacists, and the content of 1030 medication reviews (515 pre- and 515 post-) was examined. The education initiative was positively received by pharmacists and found to improve their knowledge of osteoporosis. A statistically significant increase in the number of residents for whom medication-related problems relating to osteoporosis management was identified (*χ*^2^(1,1030) = 13.476, *p* < .001) was observed following the education initiative. However, Pharmaceutical Benefits Scheme criteria, a desire to avoid polypharmacy and lack of interprofessional collaboration, were found to be barriers to these medication-related problems being resolved.

**Conclusion:**

The education initiative showed an increase in pharmacists’ knowledge and prioritisation of osteoporosis management for aged care residents. Barriers to these improvements in residents’ osteoporosis care were identified. Addressing these barriers is essential if pharmacists’ potential to enhance osteoporosis management in aged care residents is to be realised.

## Background

Residential aged care facilities (RACFs) provide accommodation and care to elderly individuals unable to live independently (Australian Institute of Health and Welfare (AIHW), 2024). Residents often experience medication-related problems, such as untreated indications, inappropriate medication selection, insufficient monitoring and incorrect dosing (Pharmaceutical Society of Australia (PSA), 2020). To address these issues and improve residents’ health outcomes, the Australian government funds initiatives to enhance pharmacists’ involvement in medication management in RACFs (Pharmacy Programs Administrator (PPA), 2024).

Osteoporosis management has been identified as an area where pharmacists may have a positive impact (PSA, 2019). A systematic literature review has demonstrated that pharmacist interventions show promise for increasing rates of osteoporosis screening, treatment initiation, and adherence to osteoporosis therapy (Laird, et al., 2023a). The 12 studies included in this review were conducted in a range of practice settings, with one completed in aged care. Interventions utilised by pharmacists tended to be multi-component in nature, involving patient education and counselling, fracture risk screening, and medication review. Pharmacists undertaking these activities in collaboration with other members of the health care team were deemed to provide greater benefit.

Over 80% of aged care residents are estimated to have osteoporosis (Australian Institute of Musculoskeletal Science (AIMS), 2022). Combined with the high fall rate of aged care residents, this leads to disproportionately high fracture rates compared to the general population (AIMS, 2022). Hip fractures, for example, occur two to 11 times more frequently, with an annual incidence of 4% (range 2.36−6%) (AIMS, 2022). Despite the availability of effective treatments, osteoporosis management is suboptimal in RACFs both in Australia and internationally (Duque et al., 2022; Lind et al., 2019; Makan et al., 2021; Niznik et al., 2021).

Limited knowledge of osteoporosis and its management among healthcare providers contribute to suboptimal care (Department of Health, Australian Government, 2019; Nguyen, 2016). For example, two recent studies found that Australian aged-care pharmacists were unfamiliar with aged-care-specific clinical guidelines and consensus recommendations, resulting in deviations from best practice (Laird et al., 2024a; Laird, Williams, et al., 2023b).

At the time of this study, the primary pharmacist clinical service undertaken in Australian RACFs was medication review , conducted under the federally funded Residential Medication Management Review (RMMR) program (Haider et al., 2021; PPA, 2024). This program involves pharmacists employed by RMMR service providers visiting facilities to perform reviews upon receipt of a referral from a General Practitioner (GP). Identified drug-related problems (DRPs) and related recommendations are then shared with the referring GP via a written report (PPA, 2024). It is encouraged (but not mandatory) that GPs provide feedback to pharmacists on these reports (Medical Benefits Schedule, 2024). After one month, pharmacists may conduct a follow-up review to address outstanding issues, incorporating GP feedback (PPA, 2024). The practice model for Australian aged-care pharmacists is currently undergoing reform. In response to the recognised need for greater access to pharmacists within RACFs, the Australian Government launched the Aged Care On-site Pharmacist (ACOP) program (Department of Health, Disability and Ageing, Australian Government, 2025). This programme funds pharmacists to be present on-site regularly, rather than visiting RACFs on an ad hoc basis, to deliver clinical services. As of 1 July 2024, RACFs have the option to continue having pharmacists conduct medication reviews under the RMMR programme or to transition to the ACOP programme.

Irrespective of the funding model under which pharmacists provide clinical services to aged-care residents, addressing pharmacists’ knowledge gaps could enhance their clinical practice. This study will pilot an osteoporosis management education initiative for aged care pharmacists and evaluate its acceptance and impact.

## Methods

### Study type

A mixed-methods pilot evaluation of an osteoporosis management education initiative for Australian aged care pharmacists was carried out between May and October 2024.

### Education initiative

The initiative was developed to equip pharmacists with knowledge and skills to address practice gaps identified in recent studies. Reference sources utilised in developing the education initiative were: ‘Prevention of Osteoporotic Fractures in Residential Aged Care: Updated Consensus Recommendations’, ‘The Royal Australian College of General Practitioners (RACGP) Osteoporosis prevention, diagnosis and management post-menopausal women and men over 50 years of age’, ‘RACGP aged care clinical guide (Silver Book), and literature on the Fracture Risk Scale assessment tool (Duque et al., 2022; GERAS Centre for Aging Research, 2021; Ioannidis et al., 2017; RACGP, 2019, 2024).

The content outline of the initiative is available in Supplemental Material 1. One researcher (CL, a female credentialed pharmacist with experience in qualitative and quantitative research, undertaking a PhD in clinical pharmacy) delivered the initiative in a live 30-minute session, attended in person or via Microsoft Teams™, followed by a question-and-answer session. Pharmacists also received two resources: Considerations when completing medication review for aged care residents to prevent osteoporotic fractures (Supplemental Material 2) and the Fracture Risk Scale: Manual Score Calculation tool (GERAS Centre for Aging Research, 2021).

## Education initiative evaluation

Evaluation followed Kirkpatrick and Kirkpatrick's (2016) four-level framework: reaction, learning, behaviour, and results. Accordingly, study objectives were: (1) to assess pharmacists’ acceptance of the initiative, (2) to evaluate awareness of osteoporosis guidelines post-education, (3) to determine if the number of identified DRPs related to fracture prevention increased, and (4) to assess whether GP acceptance of pharmacists’ recommendations improved. The final objective was chosen because GP uptake of pharmacist recommendations ultimately influences resident outcomes.

**Figure 1.**
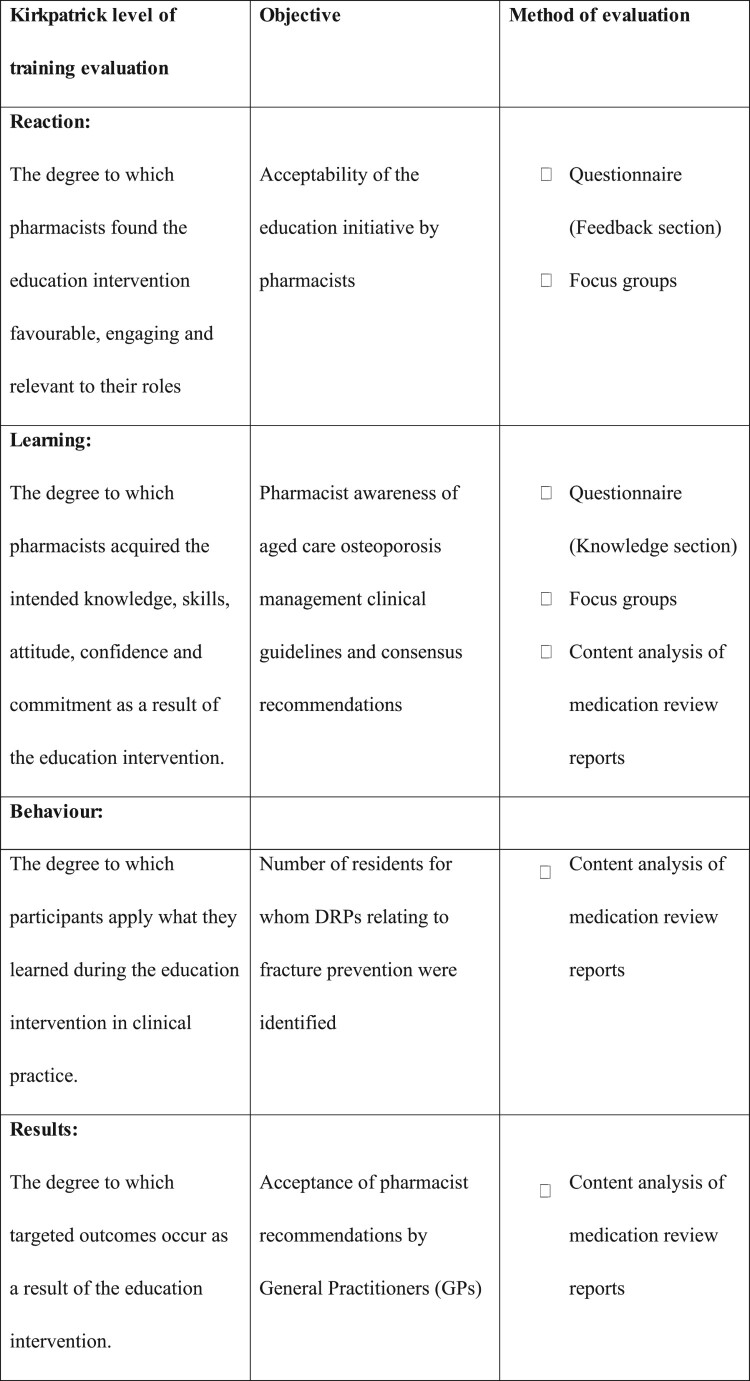
Education initiative evaluation.

Following Kirkpatrick and Kirkpatrick's (2016) recommendations, evaluations were conducted at multiple time points to assess all four levels: a questionnaire, focus groups, and content analysis of RMMR reports prepared before and after the education session (Figure 1).

### Questionnaire

Immediately after the education initiative, a questionnaire was used to capture pharmacists’ initial reaction and learning (see Supplemental Material 3). It comprised three sections: demographics, session feedback, and osteoporosis knowledge. The feedback section utilised a 5-point Likert scale (Strongly Disagree, Disagree, Neutral, Agree, Strongly Agree) to address a series of questions and three open-ended questions. The knowledge section featured multiple-choice questions based on session content. Pharmacists received a score out of 19, with one point per correct response, presented as a percentage.

It had initially been planned to administer pre- and post-education session knowledge tests. During the study design process, the timeframe available for delivering the educational session and administering the questionnaire was discussed with RMMR service providers. Due to the limited time available for these tasks, it was decided to prioritise time allocated to the education session and post-session knowledge capture; hence, the pre-test was omitted from the study design.

The questionnaire was administered via REDcap^TM^. Responses were analysed using both quantitative and qualitative methods, supported by IBM SPSS® and NVivo Pro 12® software.

### Focus groups

Eight weeks post-initiative, focus groups (facilitated by one of the researchers (CL)) explored pharmacists’ longer-term reactions and learning. This interval allowed time for implementation, GP feedback, and follow-up reviews. The question guide is available in Supplemental Material 3. Sessions were conducted via Microsoft Teams™ (access restricted to facilitator and participants) with a planned duration of 30 minutes. Thematic analysis of transcripts (generated by the transcription function in Microsoft Teams™) was completed using NVivo Pro 12® by one researcher (CL).

### Content analysis of medication review reports

Reports completed pre- and post-initiative were compared to evaluate learning, behaviour, and results. RMMR service providers submitted de-identified reports and GP acceptance data at least six weeks post-completion. This period allowed GP feedback on recommendations to be obtained. One researcher (CL) utilised a standardised form to extract residents’ age, gender, prescribed osteoporosis medicines, identified DRPs and related recommendations concerning osteoporosis, and GP acceptance of recommendations. Osteoporosis medicines were defined per the Australian Medicines Handbook (2024): antiresorptive medications (alendronate, risedronate, zoledronic acid, denosumab, raloxifene), romosozumab, teriparatide, vitamin D, and calcium. DRPs and recommendations were included if they involved osteoporosis medicines or medicines detrimental to bone health when the pharmacist specifically referred to this.

Quantitative and qualitative methods were used to evaluate the medication review reports in terms of learning, behaviour, and results, with the aid of IBM SPSS® and NVivoPro 12® software. The Chi-square test of independence was used to determine if there was any significant association between categorical variables, whilst the Mann–Whitney *U* test was used to determine if there was a significant difference between the number of osteoporosis recommendations made for each cohort. For both tests, statistical significance was set at *p*-value <0.05. The content analysis of DRPs and related recommendations was conducted in three phases. First, DRPs were classified using an adapted Hepler and Strand classification system appropriate for aged care residents (Hepler & Strand, 1990; Ruths et al., 2003) with categories for indication, effectiveness, and safety (each with sub-classifications). Second, a data-driven coding frame was developed to identify themes amongst the recommendations, using successive summarisation as outlined by Schreier (2013). Finally, the alignment of these with practice recommendations advised in the ‘RACGP aged care clinical guide (Silver Book)’ and ‘Prevention of Osteoporotic Fractures in Residential Aged Care: Updated Consensus Recommendations’ was assessed (Duque et al., 2022; RACGP, 2019). One researcher (CL) performed the coding and data analysis.

### Recruitment and sample size

A review of the literature identified that a minimum sample size of 24 participants is recommended for a feasibility study (Julious, 2005; Sim & Lewis, 2012). Recruitment of pharmacists for piloting the education initiative took place at the RMMR service provider level. Sufficient RMMR service providers were recruited to ensure the education initiative could be delivered to at least 30 pharmacists, allowing for a 20% drop-out rate. Immediately after the education initiative, individual pharmacists were invited to participate in the questionnaire and focus groups.

The sample size of medication review reports was calculated to enable detection of a 10% change in the number of residents for whom a DRP concerning osteoporosis was identified pre- and post- the education initiative. Assuming an original proportion of 50% identification of DRPs (which is the most conservative choice) and an effect size of 10%, a sample size of 515 reports at each time point was calculated.

## Results

Two RMMR service providers participated in the study. Both service providers employ multiple pharmacists. Collectively, these providers service 750 of the 2617 aged care facilities in Australia (Australian Institute of Health and Welfare, Australian Government, 2024).

**Figure 2.**
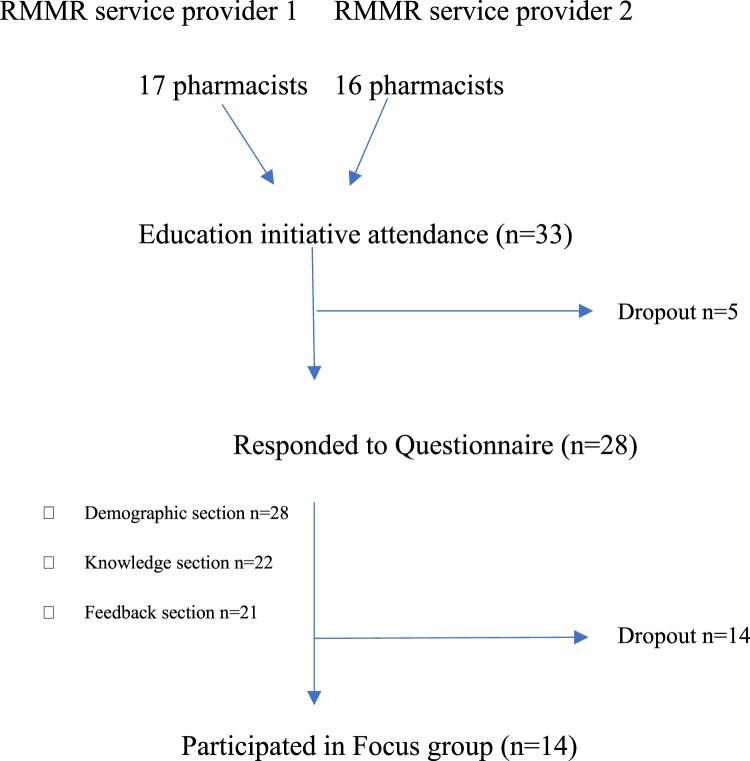
Participant flow diagram.

A seen in Figure 2, a total of 33 pharmacists attended the education initiative (Provider 1 *n* = 17, Provider 2 *n* = 16). Of these, 28 (Provider 1 *n* = 16, Provider 2 *n* = 12) responded to the questionnaire, and 14 participated in the follow-up focus groups (Provider 1 *n* = 6, Provider 2 *n* = 8).

### Questionnaire

Table 1 presents the demographics of the pharmacists who responded to the questionnaire. Six respondents only completed the demographic section.

**Table 1.** Characteristics of pharmacists who completed the questionnaire (*n* = 28).

Gender	Female	25 (89.3%)
	Male	3 (10.7%)
Level of pharmacy education	Foundation degree (BPharm/MPharm)	24 (85.7%)
	Graduate Certificate	1 (3.6%)
	Master of Clinical Pharmacy	2 (7.1%)
	Doctor of Philosophy	1 (3.6%)
Pharmacist accreditation status	Accredited	25 (89.3%)
	Undertaking accreditation	3 (10.7%)
Number of years practicing pharmacy	5–10	4 (14.3%)
	11–20	10 (35.7%)
	>20	14 (50.0%)
Number of years practicing in aged care	<5	11 (39.2%)
	5–10	8 (28.6%)
	11–20	8 (28.6%)
	>20	1 (3.6%)
Practice location of aged care facilities*	Urban	22 (78.6%)
	Regional	17 (60.7%)
	Rural	7 (25.0%)
	Remote	2 (7.1%)

* Pharmacists could select multiple responses.

The osteoporosis knowledge section of the questionnaire was completed by 22 pharmacists, with an average score of 91.9% (±7.2%).

Twenty-one pharmacists completed the feedback section of the questionnaire, which indicated that the education initiative was well received. All respondents either agreed or strongly agreed that the content was relevant to their role (agreed *n* = 3, strongly agreed *n* = 18), that the duration was appropriate for the amount of content (agreed *n* = 4, strongly agreed *n* = 17), and that they felt more confident in making recommendations about osteoporosis (agreed *n* = 7, strongly agreed *n* = 14). All except one pharmacist (who was neutral) agreed (*n* = 5) or strongly agreed (*n* = 15) that, as a result of the initiative, they would place greater priority on osteoporosis management when conducting medication reviews. Most agreed (*n* = 3) or strongly agreed (*n* = 17) that the content was suitable for their professional experience, with one neutral response.

Open-ended feedback further supports the initiative’s positive reception and educational value, as reflected in one pharmacist’s comment, ‘*It was an amazing update on this area of expertise*’ (Pharmacist 9, Provider 2). When asked about the most useful knowledge or skill(s) gained, pharmacists cited the Fracture Risk Scale (*n* = 8), evidence for monitoring and supplementing vitamin D (*n* = 5), when to commence antiresorptive medication (*n* = 4), evidence for calcium supplements (*n* = 3), increased knowledge of osteoporosis and the importance of treatment (*n* = 3), and the potential for clinical diagnosis based on minimal trauma fractures (without the need for DEXA) (*n* = 2). The most frequently reported barrier to applying knowledge was concern over doctors’ acceptance of their recommendations (*n* = 10). Other barriers included contributing to polypharmacy (*n* = 2), the cost of vitamin D (*n* = 1), and lack of access to medical history before admission (*n* = 1). One pharmacist suggested that adding more case studies could enhance the educational initiative.

### Focus groups

Two focus groups were conducted, lasting 27 and 32 minutes. As shown in Table 2, analysis of the focus group transcripts identified three main themes, each with subthemes. No diverse cases, nor minor themes were identified.

**Table 2.** Focus group themes and subthemes.

Themes	Subthemes
Improved knowledge of osteoporosis	- Greater tendency to consider osteoporosis management when completing RMMRs - Awareness that clinical diagnosis is possible without DEXA - Increased confidence in making recommendations concerning vitamin D and antiresorptive medications
Difficulties encountered in clinical practice	- Sourcing resident medical history - Incorporating FRS into their established RMMR process - Facility focus on reducing polypharmacy
Collaboration with other healthcare professions	- Need for education to be made available for other members of the healthcare team - Limited communication between the care team

Theme 1: Improved knowledge of osteoporosis

Pharmacists attending the focus groups reported positively on the education initiative, agreeing that it enhanced their knowledge of osteoporosis, which in turn influenced their clinical practice. They stated they were more likely to consider osteoporosis management during medication reviews, particularly investigating past treatment for residents with a history of fracture who was not currently receiving an antiresorptive, and ensuring that denosumab therapy was not delayed. One pharmacist’s comment highlighted this: ‘If we see fractures and no treatment, we want to find out, did they have treatment previously and it’s been ceased, or have they never had treatment? And why is that the case?’ (Pharmacist 1, Provider 2).

Understanding that osteoporosis could be clinically diagnosed (occurrence of minimal trauma fracture) without DEXA was new to some pharmacists. Supported by the Fracture Risk Scale, this realisation removed perceived barriers to treatment commencement. As commented by one pharmacist: ‘If they (residents) have had a fracture, whether hip, vertebrae or wrist, that implies they have osteoporosis, they do not need a DEXA scan. They don’t need to jump through hoops to have the treatment’ (Pharmacist 1, Provider 2).

Pharmacists agreed that the initiative clarified when to recommend vitamin D, calcium, and antiresorptive medications, boosting confidence in making recommendations. One described it as ‘clear and gave quite good direction on what to do with the information. So you go forward with confidence using it’ (Pharmacist 5, Provider 1). Pharmacists reported that they had previously received conflicting information on the use of vitamin D supplements and appreciated the clarification on this.

Theme 2: Difficulties encountered in clinical practice

Despite the positive feedback, pharmacists faced barriers in implementing the education in practice.

Accessing residents’ fracture and antiresorptive histories before admission to the RACF was challenging. Pharmacists used various methods to gather this information, such as speaking with residents, reviewing previous GP health summaries, hospital discharge summaries, and data from the residents’ My Health Record (an online health record platform developed by the Australian Government). However, this information was often incomplete, as one pharmacist remarked.

I had a new admission on bisphosphonate, and I asked them how long they've been on it for, and they don't know. And then I checked the history; there's not much there. So it's very hard to determine whether they are due for a drug holiday … (Pharmacist 3, Provider 2)

Likewise, pharmacists noted that there might be a reference to a fracture, but the details needed to meet the Australian Pharmaceutical Benefits Scheme prescribing criteria (such as the year the fracture occurred) for an antiresorptive were missing. Time constraints during RMMRs limited how thoroughly pharmacists could investigate histories: ‘...I use whatever information one can get and then leave it up to the doctor, by saying ‘I haven't been able to verify this. Please look into it further’’ (Pharmacist 2, Provider 2).

Time limitations also restricted the use of the FRS assessment tool to cases deemed highly relevant. This was reported not to be limited to the FRS assessment tool but rather the use of clinical assessment tools in general. It was suggested that incorporating the FRS into the residents’ care plans would be beneficial: ‘They have fall risk scales embedded into the care plans, and if they could write it (FRS) into that, I think it would be great’ (Pharmacist 6, Provider 1).

Avoiding polypharmacy was a high priority among pharmacists. Two issues were identified as contributing to this: the cost of medications for residents and the National Aged Care Mandatory Quality Indicator Program (QI Program) reporting requirements for residents receiving polypharmacy (Department of Health and Aged Care, Australian Government, 2025). As one pharmacist explained,

there is a lot of resistance to starting new medication. You do not want to increase medications when you are doing a medication review; you should be doing the opposite. So that is a barrier in general, but particularly in the world of polypharmacy, where facilities have to account for more than nine medications. It doesn't really help you to treat people properly. (Pharmacist 6, Provider 1)

Theme 3: Collaboration with other healthcare professions

Pharmacists recognise that optimal osteoporosis management requires a collaborative approach. Three other disciplines were identified as crucial in helping pharmacists implement the information from the education initiative: GPs, nurses, and dietitians. However, limited direct contact during medication reviews hampers pharmacists’ ability to make and follow up on recommendations. For example, pharmacists shared that they rarely had opportunities to discuss calcium intake with dietitians and found dietitians’ reports to be ‘concerned about weight, calorie intake, and protein intake. I haven't seen any specific comments about the calcium content of the diet’ (Pharmacist 5, Provider 2). Therefore, whilst pharmacists recognised that obtaining calcium through dietary sources was likely not being achieved, it appeared that this was overlooked in practice. Furthermore, pharmacists reported that most GPs do not discuss or advise on acceptance of recommendations with them, as one pharmacist commented, ‘I haven’t received any responses, so I don’t know if that’s a good thing or a bad thing’ (Pharmacist 1, Provider 1). The absence of communication following the report prevented pharmacists from addressing GP concerns regarding the implementation of recommendations.

## Content analysis of medication review reports

A total of 1030 RMMR reports were sourced from the two service providers, with 515 reports completed prior to the education initiative (Cohort 1) and 515 reports completed afterwards (Cohort 2). Table 3 presents the characteristics of the residents represented by these reports. Based on these characteristics, no statistically significant difference was found between the two cohorts.

**Table 3.** Resident characteristics prior to medication review recommendations.

Characteristic		Cohort 1 (*n* = 515)	Cohort 2 (*n* = 515)	Overall (*n* = 1030)
Demographics	Mean age in years (± SD)	85.8 (7.9)	85.6 (8.1)	85.8 (8.0)
	Female	332 (64.5%)	342 (66.4%)	674 (65.4%)
Osteoporosis medicine use	Vitamin D	323 (62.7%)	348 (67.6%)	671 (65.1%)
	Calcium	56 (10.9%)	62 (12.0%)	118 (11.5%)
	Antiresorptive/anabolic agent	127 (24.7%)	109 (21.2%)	236 (22.9%)

DRPs relating to fracture prevention were identified for 164 (31.8%) individual residents in Cohort 1 and 222 (43.1%) in Cohort 2, a statistically significant increase (*χ*^2^(1,1030) = 13.939, *p* < .001). The identified DRPs and related recommendations were sub-categorised by the medications they involved, namely, antiresorptive medications, calcium, vitamin D and medications known to have a detrimental impact on bone density. A detailed description of the identified DRPs and related recommendations is available in Supplemental Material 4, whilst Table 4 presents a summary of the number of identified DRPs and related recommendations made per resident. A statistically significant difference in the number of identified DRPs and related recommendations was observed between the two cohorts (Cohort 1 = 206, Cohort 2 = 297; U = 148878.5, *p* < .001).

**Table 4.** Number of identified DRPs and related recommendations per resident.

Number of identified DRPs and related recommendations	Cohort 1 (*n* = 515)	Cohort 2 (*n* = 515)
0	351 (68.2%)	293 (56.9%)
1	130 (25.25)	159 (30.9%)
2	26 (5.0%)	52 (10.1%)
3	8 (1.6%)	10 (1.9%)
4	0 (0%)	1 (0.2%)

Comparison of the two cohorts revealed changes in pharmacist practice regarding antiresorptive medications, calcium, and vitamin D (see Table 5).

**Table 5.** Summary of identified drug-related problems and recommendations.

Medication	Identified drug-related problem and recommendations	Cohort 1	Cohort 2
Antiresorptive medications	Undertreatment for clinical situation. Commence antiresorptive	16	35
	Potential undertreatment, complete DEXA to confirm indication and commence antiresorptive	15	23
	Unnecessary treatment. Cease antiresorptive	1	1
	Choice of antiresorptive inappropriate, change of therapy advised	2	4
	Treatment with denosumab was disrupted. Recommence	3	12
	Treatment with denosumab without recommended monitoring of serum vitamin D and calcium. Complete monitoring	10	18
	Potential drug-drug interaction involving antiresorptive. Monitor	1	0
	Dosage too high, or treatment duration too long. Change or cease	2	1
	Total	50	94
Calcium	Undertreatment for clinical situation. Commence calcium.	7	9
	Potential undertreatment, review dietary intake and commence calcium if indicated.	2	2
	Potential undertreatment, review serum level and commence calcium if indicated	7	13
	Unnecessary treatment. Cease calcium	1	5
	Contraindication for calcium supplementation. Cease calcium	1	3
	Drug interaction with calcium. Change timing of calcium administration or cease calcium	4	6
	Dose too high, or treatment duration too long. Reduce or cease	2	2
	Total	24	40
Vitamin D	Undertreatment for clinical situation. Commence vitamin D	26	36
	Potential undertreatment, review serum level and commence vitamin D if indicated	18	28
	Unnecessary treatment. Cease vitamin D.	19	5
	Contraindication identified for vitamin D, or current formulation unsuitable. Cease vitamin D or change formulation	9	14
	Dose too low. Increase dose	2	6
	Dose too high. Reduce dose	23	24
	Total	97	113

As shown in Table 5, a statistically significant increase was observed in the number of identified DRPs and related recommendations involving antiresorptive medications following the education initiative (*χ*² (1, 1030) = 15.63, *p* < .001). This was driven by an increase in pharmacists recommending the initiation of an antiresorptive, based either on clinical history or following confirmation of indication by DEXA. Additionally, following the education initiative, pharmacists were observed to have increased awareness of the dosing and potential adverse effects of antiresorptive medications, specifically denosumab, as evidenced by an increase in identifying occurrences of denosumab therapy disruptions and the administration of denosumab without the recommended monitoring of vitamin D and calcium serum levels.

Following the education initiative, a statistically significant increase in identified DRPs and related recommendations involving calcium was observed (*χ*² (1, *N* = 1030) = 4.265, *p* = .039). As shown in Table 5, this was mainly due to the increased identification of the potential need for calcium supplementation. However, pharmacists tended to suggest using serum levels rather than dietary intake assessment (as advised in clinical guidelines and consensus recommendations) to determine whether calcium supplementation was necessary.

The total number of DRPs and related recommendations involving vitamin D did not differ statistically between the two cohorts. However, the content of these recommendations did vary, with a significant reduction in pharmacists identifying unnecessary treatment with vitamin D following the education (*χ*^2^(1, *N* = 1030) = 8.361, *p* = .004) and a significant increase in pharmacists identifying a potential need to commence vitamin D (*χ*^2^(1, *N* = 108) = 4.138, *p* = .042). Hence, there was an increase in vitamin D recommendations aligning with clinical guidelines and consensus recommendations.

Recommendation acceptance data was limited, with no response recorded for 333 (86.3%) residents with an identified DRP related to osteoporosis management (Cohort 1 = 129, Cohort 2 = 204). Among the 53 (13.7%) residents with known outcomes, 28 had recommendations accepted (Cohort 1: 15, Cohort 2: 13), 15 were pending additional clinical information (such as pathology results) (Cohort 1: 10, Cohort 2: 5), and 10 were rejected (Cohort 1: 10, Cohort 2: 0). No trend was detected between recommendation acceptance and alignment with clinical guidelines.

## Discussion

Improving osteoporosis management has been identified as an area of medication management where pharmacists practising in the Australian aged care setting could potentially make a positive impact. This study piloted an educational initiative for Australian aged care pharmacists on the management of osteoporosis, which was found to be highly acceptable to participating pharmacists. Feedback from pharmacists indicated that, as a result of the initiative, they had increased knowledge of osteoporosis management and were more confident in making clinical recommendations.

Following the education initiative, an increase in the frequency of pharmacists identifying DRPs related to fracture prevention was observed among pharmacists completing medication reviews. For those DRPs involving vitamin D, an increase in pharmacists’ recommendations aligning with clinical guidelines was observed. However, there was a strong tendency for pharmacists’ recommendations regarding antiresorptive therapies and calcium supplements not to align with clinical guidelines. Whilst this finding suggests that the education initiative needs refinement to ensure pharmacists are knowledgeable on these aspects of management, data obtained from the questionnaire and focus groups raise the likelihood that factors other than pharmacists’ knowledge contributed to these recommendations. Namely, PBS prescribing criteria concerns over polypharmacy, time constraints and limited interprofessional collaboration.

Following the education initiative, pharmacists more frequently identified undertreatment and recommended initiating antiresorptive medications and calcium supplements. However, they often suggested confirmatory testing (DEXA or calcium serum levels), which deviates from the ‘RACGP aged care clinical guide (Silver Book)’ and ‘Prevention of Osteoporotic Fractures in Residential Aged Care: Updated Consensus Recommendations’ was assessed that support treatment based on clinical diagnosis and dietary assessments respectively (Duque et al., 2022; RACGP, 2024 ).

Pharmacist recommendations to use DEXA to confirm the need to initiate an antiresorptive contrast with their reports of having an increased awareness that osteoporosis can be diagnosed clinically without the need for DEXA in the questionnaire and focus groups. This discrepancy may stem from difficulties accessing fracture history. The Pharmaceutical Benefits Scheme (PBS) (2024) requires that the diagnosis of osteoporosis be confirmed either by DEXA or supporting documentation of a minimal trauma fracture for therapy to be initiated. Hence, if the necessary documentation of the minimal trauma fracture or past DEXA result is not available, referral for a current DEXA is needed to initiate therapy (RACGP, 2019). Given that it can be logistically challenging for aged care residents to undergo a DEXA scan, this finding suggests that a review of the PBS prescribing criteria for commencing antiresorptive therapy in the aged care population could improve treatment rates.

Concerns about contributing to polypharmacy may also explain deviations from guidelines. Polypharmacy is associated with increased morbidity and mortality (RACGP, 2019). Consequently, reducing polypharmacy in Australian RACFs has gained significant attention in recent years, including being one of the reporting requirements of the Australian Government's National Aged Care Mandatory Quality Indicator Program (QI Program) (Department of Health and Aged Care, Australian Government, 2025; RACGP, 2019). In the questionnaire and focus groups, pharmacists expressed a desire to avoid polypharmacy as a barrier to recommending new therapies. This aligns with findings from other studies involving pharmacists, GPs, and nurses on osteoporosis management, both specific to aged care and in the broader community, which support a strong desire amongst healthcare professionals to not increase the treatment burden, particularly of those with multiple co-morbidities and complex care needs (Cho et al., 2024; Colón-Emeric et al., 2022; Laird, et al., 2023b; Otmar, 2012; Rezae et al., 2024; Salminen et al., 2019). Therefore, pharmacists’ tendency to suggest testing to confirm indications may stem from a need for further justification when recommending the addition of medications to residents’ regimens.

Time constraints and a lack of interprofessional collaboration were also identified as barriers to pharmacists implementing the content of the education initiative. These factors are likely interconnected, with time constraints associated with the RMMR funding model preventing pharmacists from actively seeking interprofessional collaboration when performing medication reviews (Laird et al., 2023a). It is envisaged that, as pharmacists transition from the current RMMR practice model to being integrated within the facility staff under the ACOP program, there will be greater opportunity for interprofessional collaboration and time available for conducting medication reviews (Department of Health, Disability and Ageing, Australian Government, 2025).

Ultimately, for the education initiative to improve osteoporosis management, pharmacists must be proactive in making recommendations that align with clinical practice, and these recommendations must also be accepted by GPs and supported by other healthcare team members (Sadowski et al., 2020). Specifically, pharmacists highlighted the need for increased input from dietitians regarding the assessment of residents’ calcium intake, which aligns with a previous study emphasising the importance of ensuring adequate calcium intake among aged care residents (Iuliano et al., 2021). It was also suggested that fracture risk assessments be included in residents’ regular assessments, as is currently done for falls risk per the National Safety and Quality Health Standards (Australian Commission on Safety and Quality in Health Care, 2021). Furthermore, pharmacists believed that other members of the healthcare team would benefit from attending a similar educational session to ensure a shared understanding of current best practices in osteoporosis management. Expanding the education initiative to include other healthcare professionals and incorporating case studies for completion by an interprofessional team could enhance its outcomes. Additionally, the interprofessional team's involvement in such an initiative could provide an opportunity to establish agreement on who is responsible for various aspects of osteoporosis management. Noting that a recent literature review found that whilst there is enthusiasm amongst healthcare professionals for improving osteoporosis management through an interprofessional approach, consensus has not been reached for who is responsible for diagnosing and treating osteoporosis (Rezae et al., 2024).

### Strengths and limitations

To the best of the authors’ knowledge, this is the first education initiative developed and piloted that specifically targets pharmacists working in the Aged Care setting regarding osteoporosis management. The use of multiple methods to evaluate the acceptability and impact of education initiatives on clinical practice, along with piloting the education program among pharmacists who service residents from a large number of facilities in a range of geographical locations, adds to the validity of the study findings.

Despite these strengths, some limitations must be acknowledged. First, there was a high dropout rate among individual pharmacists who completed the entire questionnaire and participated in focus groups. This raises the possibility that pharmacists who did not complete the questionnaire or participate in focus groups may have held differing views on the education initiative, which could influence the study’s findings regarding the initiative's acceptability. Although the reasons for the high dropout rate among individual pharmacists are not known, it is likely the study design having pharmacists complete the questionnaire immediately following the education session contributed, whereby pharmacist time pressures to return to work duties, may have impeded participation. This could also explain the high proportion of pharmacists that did not complete all three sections of the questionnaire. Per ethics approval, the research team did not have access to individual pharmacists’ contact details, therefore, all correspondence occurred via the RMMR service providers. If the research team had individual pharmacist contact details, it would have been possible to send personal reminders to complete the questionnaire and focus groups, which may have improved participation rates. The lack of direct correspondence between the research team and individual pharmacists, however, did aid in minimising potential bias due to pre-existing relationships. Another limitation was that a single researcher (CL) delivered the education sessions, facilitated focus groups and was responsible for all data coding. Using an independent facilitator for focus groups and a second data coder would enhance the validity and reliability of the study results. Bias in the focus group participant responses could have been introduced by the facilitator being the same person as delivered the education initiative, as participants may have been less likely to make comments that could be seen as negative. It is also acknowledged that the decision not to utilise a pre-education knowledge test precluded comparison of pharmacists’ knowledge before and after the educational session. Inclusion of a pre-education session test would have been beneficial for inferring the session's impact on pharmacists’ knowledge. Finally, the only source of information used to determine GP acceptance rates of medication review recommendations was the data recorded by pharmacists which resulted in responses being received for RMMR. Involving GPs and aged care facilities in the study design would likely have identified additional data sources that could be utilised to determine GP responses to pharmacist recommendations, thereby enabling more comprehensive analysis of recommendation acceptance.

## Conclusion

This study piloted an educational programme for Australian aged care pharmacists on osteoporosis management. The initiative was well received by pharmacists and resulted in increased knowledge, confidence, and prioritisation of osteoporosis management during medication reviews. However, the low response rate from GPS to these recommendations limited the analysis of whether this led to any change in the rate of acceptance of medication review suggestions. The study identified barriers that prevented pharmacists from applying the knowledge gained through the education initiative when conducting medication reviews. These barriers include the PBS criteria for initiating antiresorptive therapies, a desire to avoid polypharmacy, time constraints during medication reviews, and a lack of interprofessional collaboration. Overcoming these barriers is crucial if the pharmacist education programme is to effectively improve osteoporosis management for aged care residents.

## Supplementary Material

Supplemental Material
