# Supplementary material for: Equipping pharmacists to improve osteoporosis management for aged care residents: a pilot study
Source: J Pharm Policy Pract. 2026 May 21;19(1):2669623. doi: 10.1080/20523211.2026.2669623 (PMC13195720; doi:10.1080/20523211.2026.2669623)
Supplement: Supplemental Material [file JPPP_A_2669623_SM4284.docx]

**Supplementary Online Materials 1**

**Education initiative content outline**

**Learning objectives:**

1. To be able to define osteoporosis.
2. To be able to identify risk factors for osteoporosis.
3. To describe the impact of osteoporosis on aged care residents.
4. To be able to explain how osteoporosis is diagnosed.
5. To understand the role of fracture risk assessments in identifying residents who would benefit from osteoporosis medicines.
6. To describe the mechanism of action of osteoporosis medicines
7. To be familiar with the content of the Australian “Prevention of Osteoporotic Fractures in Residential Aged Care: Updated consensus recommendations”
8. To be able to implement the “Prevention of Osteoporotic Fractures in Residential Aged Care: Updated consensus recommendations” to guide osteoporosis management for aged care residents.

**Content:**

***Module one- Osteoporosis essentials***

- Bone physiology and anatomy
- Normal and osteoporotic bone
- Risk factors for osteoporosis
- Fractures arising from osteoporosis.
- Impact of osteoporosis on aged care residents

***Module two- Diagnosing osteoporosis.***

- Measuring bone mineral density (BMD) using Dual-energy X-ray absorptiometry (DEXA)
- Fracture risk assessments: the Fracture Risk Scale
- Case study: Completing a fracture risk assessment using the Fracture Risk Scale manual calculation form.

***Module three – Osteoporosis medicines***

- Nutritional supplements
- Antiresorptive therapies
- Anabolic agents

***Module four- Pharmacist role in preventing osteoporotic fractures in aged care***

- Falls prevention.
- Use of nutritional supplements
- Use of antiresorptive therapies and anabolic agents

**Supplementary online materials 2**

**Supplementary Online Materials 3**

**Questionnaire**

Opening statement: Thank you for completing this questionnaire following participation in the Osteoporosis Management for Aged Care residents’ education session. The questionnaire consists of questions regarding demographics, knowledge of osteoporosis management in aged care, and evaluation of the education session. Your feedback is important.

***Pharmacist Demographics***

1. What is your gender?
   1. Male
   2. Female
   3. Non-binary
   4. Prefer not to answer
2. How many years have you been practicing as a registered pharmacist?
   1. <5 yrs
   2. 5-10 yrs
   3. 11-20 yrs
   4. >20 years
3. Please indicate your highest level of education relating to pharmacy.
   1. Bachelor of Pharmacy or Master of Pharmacy
   2. Graduate certificate
   3. Graduate diploma
   4. Master of Clinical Pharmacy
   5. PhD
   6. Other: _____________________
4. Are you accredited to complete medication reviews?
   1. No
   2. Yes
   3. Currently undertaking accreditation
5. Please indicate the number of years you have provided pharmacy services to aged care residents.
   1. _________
6. Where are the Residential Aged Care facilities you service located? (Select all that apply)
   1. Urban
   2. Regional
   3. Rural
   4. Remote

***Osteoporosis knowledge***

1. Please indicate which of the following is a risk factor for osteoporotic fractures in aged care residents. Mark all that apply.
   1. Female sex
   2. History of previous fractures
   3. History of falls
   4. High body weight
   5. Psychotropic medication use
   6. Bowel or bladder incontinence
   7. Non-ambulant
   8. Low serum vitamin D
2. Please indicate which medications are associated with a negative impact on bone health, leading to increased fracture risk. Mark all that apply
   1. Oral glucocorticoids
   2. Sodium valproate
   3. Thiazolidinediones
   4. Metformin
   5. Esomeprazole
   6. Anastrozole
   7. Sertraline
   8. Tamoxifen
3. Based on the World Health Organization (WHO) definition of osteoporosis, which T-score represents someone with osteoporosis?
4. T-score= -1.0
5. T-score= +2.5
6. T-score= -1.5
7. T-score= -2.7
8. Which of the following statements best describes the recommended daily calcium intake for aged care residents?

a) A total daily intake of 600mg

b) A total daily intake of 1000mg (up to 600mg from calcium supplements)

c) A total daily intake of 1300mg (up to 800mg from calcium supplements)

d) A total daily intake of 1300mg (up to 600mg from calcium supplements)

1. Which of the following serum 25(OH) levels is considered optimal for aged care residents?

a) >25nmol/L

b) >50nmol/L

c) >75noml/L

d) >100nmol/L

1. Please indicate in which circumstances monitoring of serum vitamin D is recommended. Mark all that apply
2. Serum vitamin D should be monitored for all residents, as they are all at high risk of deficiency
3. Serum vitamin D should be monitored for residents at high falls risk
4. Serum vitamin D should be monitored for residents who have sustained a new fracture
5. Serum vitamin D should be monitored for residents who are receiving antiresorptive therapy
6. Mary is an 85-year-old female who has recently been admitted to an aged care facility and referred for a medication review. She was transferred to the aged care facility from a hospital where she had been admitted for management of a distal radius fracture resulting from a fall. Most recent pathology includes an eGFR=31mL/min.

Mary’s current medications are:

Aspirin 100mg 1 mane

Perindopril 4mg 1 nocte

Esomeprazole 20mg 1 nocte

Metoprolol 50mg ½ BD

Frusemide 40mg 1 mane

Paracetamol 500mg 2 tds and 2 PRN

Docusate and senna 50mg/8mg 2 nocte

Based on the information available, please select which of the following is the most appropriate course of action:

1. Mary should be referred for a DEXA to confirm a diagnosis of osteoporosis. If osteoporosis is confirmed, Mary should be commenced on a bisphosphonate.
2. Mary should be referred for a DEXA to confirm a diagnosis of osteoporosis. If osteoporosis is confirmed, Mary should be commenced on denosumab.
3. Mary can be diagnosed with osteoporosis based on clinical presentation. Mary should be commenced on either a bisphosphonate or denosumab.
4. Mary can be diagnosed with osteoporosis based on clinical presentation. Mary should be commenced on denosumab.

***Feedback on the education session***

1. The content covered in this course is relevant and applicable to my role.

םStrongly disagree, ם Disagree, ם Neutral, ם Agree, ם Strongly agree

1. The course length was sufficient for the amount of content.

ם Strongly disagree, ם Disagree, ם Neutral, ם Agree, ם Strongly agree

1. The course content was appropriate for my professional experience.

ם Strongly disagree, ם Disagree, ם Neutral, ם Agree, ם Strongly agree

1. As a result of this education session, I will place greater priority on osteoporosis management when undertaking medication reviews.

ם Strongly disagree, ם Disagree, ם Neutral, ם Agree, ם Strongly agree

1. As a result of this education session, I am more confident in making recommendations regarding osteoporosis management for aged care residents.

ם Strongly disagree, ם Disagree, ם Neutral, ם Agree, ם Strongly agree

1. What knowledge or skills(s) presented in this education session will be most helpful in providing pharmacy services to aged care residents?

___________________________________________________________________________

___________________________________________________________________________

1. What, if any, barriers can you perceive to applying what you learned?

___________________________________________________________________________

___________________________________________________________________________

1. Please share any other comments or recommendations you may have for improving this course.

___________________________________________________________________________

___________________________________________________________________________

**Supplementary Materials 3**

**Focus group question guide**

Opening statement: This focus group is an opportunity to reflect upon the recently attended education session on osteoporosis management for aged care residents. The purpose of this focus group is to provide feedback on this education session. There are no right or wrong answers. Your willingness to engage honestly is greatly appreciated, as is your respect for the opinions and experiences of all participants in this session. You are asked to hold the comments voiced in this session in confidence. This focus session will be recorded to aid the research team in analysing the feedback received.

1. How are you currently using what you learned during the osteoporosis management for aged care resident education session?
2. What positive outcomes are you seeing as a result of what you are doing?
3. Have you encountered any barriers to implementing the consensus recommendations? If so, what are they?
4. Have you utilised the fracture risk assessment tool (Fracture Risk Scale) since the training? If yes, could you describe your experiences with using this tool? If no, what has prevented you from using this tool?
5. Are there any elements of osteoporosis management for aged care residents you would like further support with? How would you like this support to be provided to you?

**Supplementary Online Materials 4**

Drug-related problems and recommendations relating to fracture prevention

| Drug-related problem | | Summary of DRP and recommendation | Align with clinical guidelines and consensus recommendations | Pre-education initiative | Post-education initiative |
| --- | --- | --- | --- | --- | --- |
| Indication | Need for an additional drug: undertreatment for diagnosis | Vitamin D: | | | |
|  |  | Resident not prescribed vitamin D. Vitamin D deficiency is common in aged care residents. Commence supplement | Yes | 16 | 24 |
|  |  | Resident not prescribed vitamin D. Vitamin D deficiency has been detected. Commence supplement. | Yes | 4 | 7 |
|  |  | Resident receiving an antiresorptive and not prescribed vitamin D. Vitamin D should be supplemented to achieve the clinical effect of antiresorptive. Commence supplement | Yes | 6 | 5 |
|  |  |  | | | |
|  |  | Calcium: | | | |
|  |  | Resident with history of osteoporosis or high risk of fracture is not prescribed calcium. Adequate calcium is necessary to reduce risk of fracture. Commence supplement. | No | 5 | 7 |
|  |  | Resident not prescribed calcium and dietary intake is inadequate. Adequate calcium is necessary to reduce risk of fracture. Commence supplement. | Yes | 1 | 0 |
|  |  | Resident prescribed vitamin D without calcium. Vitamin D is only of benefit if combined with adequate calcium intake. Commence calcium supplement. | No | 0 | 1 |
|  |  | Resident is hypo calcaemic and not receiving a calcium supplement. Commence calcium supplement and monitoring of serum level advised. | Yes | 1 | 1 |
|  |  |  | | | |
|  |  | Antiresorptive: | | | |
|  |  | Resident with history of osteoporosis not prescribed an antiresorptive. Commence antiresorptive. | Yes | 14 | 31 |
|  |  | Resident with history of osteoporosis not prescribed an antiresorptive. Review past medical history and if not on a bisphosphonate drug holiday, commence antiresorptive. | Yes | 2 | 4 |
|  |  |  | | | |
|  | Need for diagnostic test: unclear or not confirmed indication; need for review | Vitamin D: | | | |
|  |  | Resident not prescribed vitamin D. Vitamin D deficiency is common in aged care residents. Obtain serum level, and supplement if indicated. | Yes | 12 | 13 |
|  |  | Resident has osteoporosis/high risk of fracture and is not prescribed vitamin D. Obtain serum level and supplement if indicated. | Yes | 6 | 15 |
|  |  |  | | | |
|  |  | Calcium: | | | |
|  |  | Resident has osteoporosis/high risk of fracture and is not prescribed calcium. Obtain serum level and supplement if indicated. | No | 7 | 13 |
|  |  | Resident has osteoporosis/high risk of fracture and is not prescribed calcium. Review dietary intake and supplement if indicated. | Yes | 2 | 2 |
|  |  |  | | | |
|  |  | Antiresorptive: | | | |
|  |  | Resident not prescribed antiresorptive and at risk of osteoporosis. Refer for DEXA and commence antiresorptive if indicated. | Yes | 9 | 10 |
|  |  | Resident is not prescribed antiresorptive and has a past diagnosis of osteoporosis or a history of minimal trauma fracture. Refer for DEXA and commence antiresorptive if indicated. | No | 4 | 12 |
|  |  | Resident with a history of osteoporosis is not prescribed an antiresorptive. Perform fracture risk assessment. Commence an antiresorptive if indicated | Yes | 0 | 1 |
|  |  | Resident has a history of osteoporosis, currently having a bisphosphonate drug holiday. Refer for DEXA and commence antiresorptive if indicated. | Yes | 1 | 0 |
|  |  | Resident has a history of osteoporosis, previously prescribed denosumab. Refer for DEXA and recommence antiresorptive if indicated. | Yes | 1 | 0 |
|  |  |  | | | |
|  | Unnecessary treatment: no appropriate medical indication; therapeutics or pharmacological duplication; drugs used for the treatment of avoidable adverse drug reactions | Vitamin D: | | | |
|  |  | Resident is prescribed vitamin D. Vitamin D supplementation provides no benefit unless the resident has frank deficiency. Cease vitamin D. | No | 16 | 3 |
|  |  | Resident is prescribed vitamin D. Resident is non-ambulant with low fracture risk or is palliative. There is limited clinical benefit from ongoing vitamin D. Cease vitamin D. | Yes | 3 | 1 |
|  |  | Resident prescribed both cholecalciferol and calcitriol formulations of vitamin D. This represents duplicated therapy and increases the risk of hypercalcemia. Obtain serum level, review indication, and cease one formulation as clinically indicated. | Yes | 0 | 1 |
|  |  |  | | | |
|  |  | Calcium: | | | |
|  |  | Resident is prescribed calcium. Resident is non-ambulant with low fracture risk or is palliative. There is limited clinical benefit for ongoing calcium. Cease calcium | Yes | 1 | 1 |
|  |  | Resident is prescribed calcium. Calcium supplements are only of benefit if dietary intake is insufficient. Review dietary intake and cease if able. | Yes | 0 | 1 |
|  |  | Resident is prescribed both calcium and vitamin D. Use of vitamin D negates the need for a calcium supplement. Cease calcium. | No | 0 | 2 |
|  |  | Resident is prescribed calcium without vitamin D. Monotherapy with calcium provides no benefit. Cease calcium. | No | 0 | 1 |
|  |  |  | | | |
|  |  | Antiresorptive: | | | |
|  |  | Resident is prescribed an antiresorptive. Resident is non-ambulant with low fracture risk or is palliative. There is limited clinical benefit for ongoing antiresorptive. Cease antiresorptive. | Yes | 0 | 1 |
|  |  | Resident is prescribed an antiresorptive, however, has no documented history of osteoporosis. Refer to DEXA and cease antiresorptive if appropriate. | Yes | 1 | 0 |
|  | |  | | | |
| Effectiveness | Choice of drug: drug not indicated for condition; more effective drug available; contraindication present | Vitamin D: | | | |
|  |  | Resident has swallowing difficulties and using a formulation of vitamin D that can not be crushed. Change of formulation will aid adherence. Change to liquid/crushable formulation. | Yes | 8 | 10 |
|  |  | Resident has a high daily pill burden, including daily vitamin D. Weekly vitamin D administration is equally effective. Change to weekly vitamin D to reduce pill burden and improve adherence. | Yes | 1 | 3 |
|  |  | Resident has renal failure and prescribed cholecalciferol. The ability to convert cholecalciferol to active form is likely reduced. Review serum level and change to calcitriol if required. | Yes | 0 | 1 |
|  |  |  | | | |
|  |  | Calcium: | | | |
|  |  | Resident prescribed calcium and is hypercalcaemic/experiencing adverse effects. Cease calcium and monitor serum level. | Yes | 1 | 3 |
|  |  |  | | | |
|  |  | Vitamin D and calcium: | | | |
|  |  | Resident prescribed separate formulations of vitamin D and calcium and has a complex medication regimen. Change to a combination formulation to reduce pill burden and aid adherence. | Yes | 3 | 3 |
|  |  |  | | | |
|  |  | Antiresorptive: | | | |
|  |  | Resident prescribed an oral bisphosphonate, and has swallowing difficulties. Review the ongoing need for antiresorptive, with a view to either a drug holiday or change to denosumab or zoledronic acid. | Yes | 2 | 4 |
|  |  |  | | | |
|  | Dosage too low | Vitamin D: | | | |
|  |  | Prescribed a vitamin D supplement; however, there is clinical reason to suspect the current dose may not be sufficient. Review serum level. Dose increase vitamin D if serum level is <75nmol/L. | Yes | 2 | 4 |
|  |  | High dose intermittent vitamin D prescribed. High-dose intermittent dosing regimens are not recommended. Change to weekly dosing regimen. | Yes | 0 | 1 |
|  |  | Vitamin D 10,000iU every 3 months is prescribed. This dose appears low, potentially intended to be 100,000iU. Review dose. | No | 0 | 1 |
|  |  |  | | | |
|  |  | Antiresorptive: | | | |
|  |  | Resident previously prescribed denosumab. Therapy has been disrupted. Recommence denosumab. | Yes | 3 | 12 |
|  | |  | | | |
| Safety | Risk for single adverse drug reactions: unfavourable safety profile | Antiresorptive: | | | |
|  |  | Resident prescribed denosumab, which increases risk of hypocalcaemia. Vitamin D and calcium have not been monitored. Review serum vitamin D and calcium prior to administration, and replete if required. | Yes | 10 | 18 |
|  |  |  | | | |
|  |  | Non-osteoporosis medication: | | | |
|  |  | Resident taking a medication that can increase risk of fracture. Reduce dose of this medication. | Yes | 18 | 35 |
|  |  | Resident taking a medication that can increase risk of fracture. Refer for DEXA and commence antiresorptive if indicated. | Yes | 4 | 2 |
|  |  | Resident taking a medication that can increase risk of fracture. Reduce dose of this medication and refer for DEXA and commence antiresorptive if indicated. | Yes | 5 | 3 |
|  |  | Resident taking a medication that can increase risk of fracture. Reduce dose of this medication, obtain serum vitamin D and calcium and supplement if indicated, refer for DEXA and commence antiresorptive if indicated. | Yes | 4 | 5 |
|  |  | Resident taking a medication that can increase risk of fracture. Commence calcium. | No | 1 | 1 |
|  |  | Resident taking a medication that can increase risk of fracture. Commence vitamin D. | Yes | 0 | 1 |
|  |  |  | | | |
|  | Drug-drug interaction | Calcium: | | | |
|  |  | Resident prescribed calcium to be administer with a medication that can have absorption impaired by calcium. Adjust the administration regimen to separate these medications. | Yes | 4 | 5 |
|  |  | Resident prescribed calcium in combination with calcitriol. This combination is not recommended due to increased risk of hypercalcaemia. Cease calcium and monitor serum level. | Yes | 0 | 1 |
|  |  |  |  | | |
|  |  | Antiresorptive: | | | |
|  |  | Resident receiving denosumab and immunosuppressant. Denosumab may increase immunosuppression. Monitor and consider prophylactic antibiotics. | No | 1 | 0 |
|  |  |  | | | |
|  | Dosage too high; excessive treatment duration | Vitamin D: | | | |
|  |  | Prescribed vitamin D >1000iU daily. Serum level confirmed deficiency corrected. Reduce dose to 1000iU daily. | Yes | 8 | 8 |
|  |  | Prescribed vitamin D >1000iU daily. It is likely deficiency is now corrected. Obtain serum level and reduce dose to 1000iU daily if able. | Yes | 11 | 9 |
|  |  | Prescribed vitamin D >1000iU daily. It is likely deficiency is now corrected. Reduce dose to 1000iU daily. | No | 4 | 7 |
|  |  |  | | | |
|  |  | Calcium: | | | |
|  |  | Resident prescribed calcium >600mg daily. Clinical guidelines advise a maximum daily dose of 600mg daily. Reduce dose to 600mg daily | Yes | 2 | 1 |
|  |  | Resident was prescribed calcium for hypocalcaemia. It is likely deficiency is now corrected. Obtain serum level and reduce if able. | Yes | 0 | 1 |
|  |  |  | | | |
|  |  | Antiresorptive: | | | |
|  |  | Resident has been receiving oral bisphosphonate for over 5 years. Drug holiday advised. | Yes | 1 | 0 |
|  |  | Resident prescribed denosumab for osteoporosis. Dosing regimen charted is not consistent with recommended dose. Correct chart. | Yes | 1 | 1 |
